# The potential of Quercetin to protect against loperamide‐induced constipation in rats

**DOI:** 10.1002/fsn3.2296

**Published:** 2021-05-04

**Authors:** Wenhui Liu, Aimin Zhi

**Affiliations:** ^1^ Fujian Fengjiu Biotechnology Co., Ltd. Zhangzhou China

**Keywords:** constipation, loperamide, Quercetin, rat, signal pathways

## Abstract

Constipation is the most common gastrointestinal complaint all over the world, and it is a risk factor of colorectal cancer. In this study, the protective of Quercetin against loperamide‐induced constipation and its potential mechanism in a rat model were investigated. Results showed that Quercetin at 25 mg/kg and 50 mg/kg could significantly (*p* < .05) increase the intestinal transit rate, motilin, gastrin, substance P levels, and concentration of short‐chain fatty acids (SCFAs), reduce the somatostatin levels, and improve the gastrointestinal peristalsis of rats. In addition, the expression levels of enteric nerve‐related factors, glial cell line‐derived neurotrophic factor (GDNF), transient receptor potential vanilloid 1 (TRPV1), nitric oxide synthase (NOS), c‐Kit, stem cell factor (SCF), and aquaporin 3 (AQP3) were examined by RT‐qPCR and/or Western blot analysis. The results suggest that Quercetin relieves loperamide‐induced constipation by increasing the levels of interstitial cells of Cajal markers (c‐Kit and SCF), as well as AQP3. In conclusion, the present study suggested that Quercetin exerted a protective effect against loperamide‐induced constipation, which may be associated with its role in regulation of multiple signal pathways.

## Graphical abstract

Quercetin exerted a protective effect against loperamide‐induced constipation. The possible protective mechanisms of Quercetin might involve the innate immune response via modulating the c‐kit/SCF pathway and other pathways.

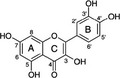

## 1 INTRODUCTION

Constipation is a prevalent and burdensome gastrointestinal (GI) disorder that is highly prevalent and seriously affects the quality of life (Huang et al., 2020). It is characterized by slowing of gut transit time, harder stool, and infrequent defecation (Hayeeawaema et al., 2019). Constipation can cause an increase in the amount of harmful microorganisms in the intestine, thereby destroying the tissue of the small intestine and causing damage to the intestinal wall and the intestinal villi, such that intestinal peristaltic function is also greatly affected, which can cause or aggravate constipation (Zhang et al., 2018; Zhao et al., 2018). In addition to affecting normal bowel movements, the long‐term accumulation of toxic substances in the body will cause other intestinal diseases (such as colorectal cancer, irritable bowel syndrome, and some other gastrointestinal illnesses) (Zhang et al., 2018). Meanwhile, the pathogenesis of constipation has not been fully understood and believed to be multifactorial (Lin et al., 2015). Dietary fiber, antibiotic, and purgative were the part of the regime in sows (Mccormack et al., 2018). Addition of dietary fiber increases fecal mass and colonic transit time (Yang et al., 2012). Laxatives often cause many side effects including diarrhea, upset stomach, vomiting, and stomach cramping. In particular, osmotic laxatives containing poorly absorbable ions such as magnesium or phosphate can cause metabolic disturbances particularly in the presence of renal impairment (Chen et al., 2020, 2020).

**FIGURE 1:**
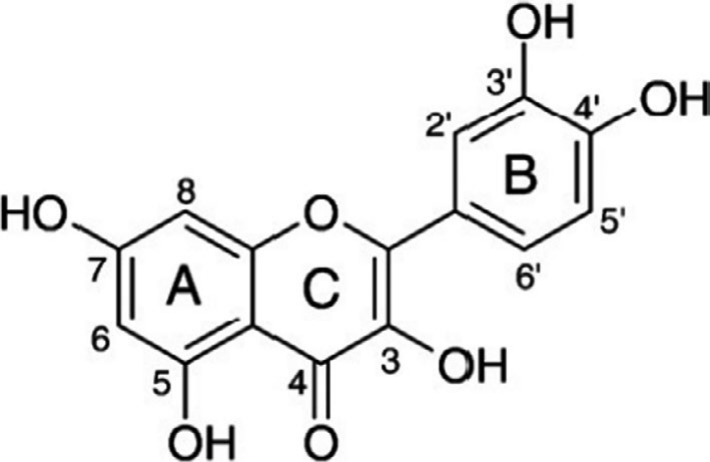
Molecular structure of Quercetin

Quercetin (QCT) is an abundant flavonoid found in many fruits, vegetables, and grains. Structurally, they have an oxygen‐containing ring between two benzene rings (Figure 1), due to which they exist in several different forms in human body, such as QCT glycoside, QCT sulfate, QCT glucuronide, and methylated QCT (Kim et al., 2018). QCT is claimed to exert beneficial health effects protection against various diseases, such as osteoporosis, certain forms of cancer, pulmonary, and cardiovascular diseases (Boots et al., 2008). But, there is scant evidence for a direct role of QCT on gastrointestinal diseases such as diarrhea, vomiting, gastroenteritis, constipation, irritable bowel syndrome (IBS), and celiac disease. Several reports on extracts and compounds containing flavonoids have provided evidences for the possibility that flavonoid contributes to the improvement of constipation. Although previous studies have provided some information regarding the possibility of a correlation between some intestinal bowel diseases and QCT, little studies have investigated the laxative effects of QCT in the constipated animal model (Kim et al., 2018; Yu et al., 2016).

In the present study, a rat constipation model was established using loperamide to observe the constipation‐inhibiting effect of the QCT. By detecting the relevant indicators in serum and small intestinal tissue, the recovery of intestinal function by QCT in constipated rats was verified. Additionally, gene expression in the small intestine was determined by RT‐qPCR to further elucidate the molecular mechanism of QCT in relieving constipation. The experimental results will provide reference data for subsequent industrialization and application for better utilization of QCT in the pharmaceutical industry.

## 2 MATERIALS AND METHODS

### 2.1 Animals and reagents

Healthy male *SD* rats (SPF grade, 180–220 g) were purchased from Shanghai Jiesijie Laboratory Animal Co., Ltd (Certificate no. SCXK20130006). All protocols of animal experiments were approved by the Institutional Animal Care and Use Committee at Jiangnan University (No. No20180115b1920520). The procedures were performed in accordance with the Guidance of the Care and Use of Laboratory Animals in China. Quercetin was obtained from Shanghai Tauto Biotech Co., LTD (Shanghai, China).

### 2.2 Experimental design

All rats were housed under controlled environment (room temperature of 23 ± 2°C with 12/12 hr light‐dark cycles) and provided with water and normal chow diet ad libitum. After 1 week of acclimatization, the rats were randomly divided into five groups (*n* = 8): Ctrl, LOP, LQCT (low dose QCT), MQCT (Middle dose QCT), and HQCT (high dose QCT). The loperamide‐induced constipation rat model was established according to previous study (Lee et al., 2012; Lee et al.; 2020; Li et al., 2021; Wang et al., 2020). The rats of LOP group and three QCT groups were subcutaneously injected with loperamide (3 mg/kg∙body weight, dissolved in saline) twice a day for 3 days, and the rats of Ctrl group were injected with the same volume of saline. Next, three QCT treated groups were orally administered 10, 25, or 50 mg/kg body weight QCT at once, while the Lop group received the same volume of 1× PBS under the same pattern. At 24 hr after QCT treatment, all *SD* rats were euthanized using CO_2_ gas, after which tissue samples were acquired and stored in Eppendorf tubes at −70°C until assay.

### 2.3 Analysis of feeding behavior and excretion parameters

Feeding behaviour and excretion parameters were measured as previously described (Kim et al., 2016, 2018). The food weight, water volume, and body weight of *SD* rats were tested daily at 10:00 a.m. throughout the experimental period using an electrical balance (for food and body weight) and a measuring cylinder (for water volume). The average food intake and water consumption were then calculated. In addition, feces of *SD* rats in different groups were harvested. The feces number and weight were also measured. The feces water content was also analyzed using the following formula:

$$\text{Feces water content}=\left(A‐B\right)/A\times 100$$

Where *A* is the weight of fresh feces collected from *SD* rats of subset groups and *B* is the weight of stools after drying at 60°C for 12 hr. All measurements were performed three times to ensure accuracy.

### 2.4 Gastrointestinal transit ratio

Charcoal meal test is widely used for the measurement of gastrointestinal transit in rodents (Chen et al., 2020, 2020). At the end of experimental day, the rats were fasted from food for 24 hr (water was provided). The *SD* rats were fed with 1 ml of charcoal meal (3% suspension of activated charcoal in 0.5% aqueous methylcellulose) as previously described (Choi et al., 2014). Wait at least 30 min of peristalsis, the rats were euthanized. And then, the stomach and intestine were coharvested to observe the transit distance of charcoal meal. Gastrointestinal transit rate was calculated as equation:

Gastrointestinal transit rate (%)=[distance traveled by the charcoal (cm)/whole length of the small intestine (cm)]×100

### 2.5 Measurement of short‐chain fatty acids (SCFAs)

Short‐chain fatty acids (SCFAs) were measured using the previous method (Chen et al., 2020, 2020; Wang, et al. 2017; Wang, Hu, et al., 2017). Briefly, 50 mg fecal sample was soaked with saturated sodium chloride, acidified with 5% (v/v) H_2_SO_4_, and then extracted with diethyl ether. For analysis, the sample was injected into a gas chromatography‐flame ionization detector equipped with an HP‐INNOWAXax GC column (Agilent, Santa Clara, CA, USA). Detection conditions were as follows: initial and final column oven at 120°C for 1 min and 200°C for 5 min, respectively, injection and detection column temperature was 250°C, and the flow rate was 1.85 ml/min. Acetic acid, propionic acid, and butyric acid contents were used as standards.

### 2.6 Detection of gastrointestinal hormone content

To determine the serum levels of gastrin (Gas), motilin (MTL), somatostatin (SS), substance P (SP), vasoactive peptide (VIP), and acetylcholine esterase (AChE) in rats, the plasma was allowed to rest for 1 hr. After that, the plasma was centrifuged at 2,200 g for 15 min. The serum levels of Gas, MTL, SS, SP, VIP, and AChE in rats were determined by kits (Nanjing Jiancheng Bioengineering Institute, Nanjing, China) according to the manufacturer's instructions.

### 2.7 Count of viable bacteria in feces

Fresh feces were collected and stored in a refrigerator at −20°C for viable bacteria count. 0.5 g of fresh feces were added into 50 ml saline using the sterile cotton swab and shaken for 30 min. Serial dilutions of samples were inoculated on MRS medium, TPY medium, EMB medium, and EC medium. After anaerobic (MRS, TPY) and aerobic (EMB, EC) culture at 37°C for 48–72 hr, the number of viable bacteria (CFU/g) of *Lactobacillus*, *Bifidobacterium*, *Enterobacter,* and *Enterococcus* per gram of feces was calculated.

### 2.8 Reverse transcription‐polymerase chain reaction (RT‐qPCR)

Total RNA was isolated from the small intestine tissue according to the manufacturer's instructions using Trizol reagent (Invitrogen), and its reverse transcription was performed according to previous reports (Jia et al., 2020). PCR primer sequences of the genes were shown in Table 1. Real‐time PCR was performed on a LightCycler^®^ Nano thermal cycler (Roche Applied Science, Penzberg, Germany) using a SYBR Green FastStart kit (Roche, Basel, Switzerland). PCR cycles are as follows: 1 cycle at 50°C for 2 min, 1 cycle at 95°C for 10 min, followed by 40 cycles at 95°C for 15 s, and at 60°C for 1 min. Relative expression was calculated using the 2^−ΔΔCt^ method.

**TABLE 1:** Sequences of primers used in this study

Gene Name	Sequence
c‐Kit	Forward: 5′‐AGACCGAACGCAACT‐3′
	Reverse: 5′‐GGTGCCATCCACTTCA‐3′
SCF	Forward: 5′‐AAACTGGTGGCGAATC‐3′
	Reverse: 5′‐CACGGGTAGCAAGAAC‐3′
GDNF	Forward: 5′‐TTTTATTCAAGCCACCAT‐3′
	Reverse: 5′‐AGCCCAAACCCAAGTCA‐3′
TRPV1	Forward: 5′‐AGCGAGTTCAAAGACCCAGA‐3′
	Reverse: 5′‐TTCTCCACCAAGAGGGTCAC‐3′
iNOS	Forward: 5′‐AGAGAGATCGGGTTCACA‐3′
	Reverse: 5′‐CACAGAACTGAGGGTACA‐3′
AQP3	Forward: 5′‐ATCATGGAGGATGACGAGTT‐3′
	Reverse: 5′‐GCCATCCACTTCACAGGTAG‐3′
GAPDH	Forward: 5′‐AGGTCGGTGTGAACGGATTTG‐3′
	Reverse: 5′‐GGGGTCGTTGATGGCAACA‐3′

### 2.9 Western blotting analysis

Total homogenate proteins were extracted from the intestine tissue of different groups using the Cell lysis buffer for Western and IP (Beyotime). Following centrifugation at 11,400 g for 5 min, the protein concentrations were determined using a BCA Protein Assay Kit (Beyotime). The proteins in each sample were determined by sodium dodecyl sulfate polyacrylamide gel electrophoresis, transferred to a polyvinylidene difluoride membrane, and exposed to an appropriate antibody (the dilution rates are as follows: anti‐ c‐Kit, anti‐AQP3, and anti‐β‐actin, 1:1,000) (Beyotime). The proteins were visualized by using an enhanced chemiluminescence detection system (Amersham Biosciences) with a horseradish peroxidase conjugated anti‐rabbit secondary antibody. Images were acquired using a ChemiDoc MP Imaging System (Bio‐Rad).

### 2.10 Statistical analysis

Statistical package for social sciences (SPSS) software version 22.0 (SPSS Inc.) was used to perform statistical analysis. The significance of differences among data was assessed using the ANOVA program.

## 3 RESULTS

### 3.1 Effect of QCT treatment on feeding behavior and excretion parameters

Feeding behavior and excretion parameters (such as body weight, food intake, water consumption, fece number, fece weight, and fece water content) were examined in Lop‐induced constipated *SD* rats. After course of loperamide treatment, the water content of feces from rats in the model group is significantly different from that in the normal group (*p* < .05), suggesting that treatment reduced the water content and resulted in drier feces. This effect would increase the difficulty of defecation, similar to the clinical symptoms of constipation. No significant differences in the body weight and food intake were observed, although water consumption decreased in the MQCT and HQCT groups (Table 2). However, the fece number, fece weight, and fece water content of *SD* rats in MQCT and HQCT group were improved when compared with those in LOP group, suggesting that the QCT facilitated defecation in rats with loperamide‐induced constipation.

**TABLE 2:** Alteration on the constipation parameters after QCT treatment

Category	Ctrl	LOP	LQCT	MQCT	HQCT
Body weight (g)	306.2 ± 19.3	299.5 ± 12.6	301.9 ± 13.7	305.8 ± 16.2	306.8 ± 14.3
Food intake (g/day)	24.68 ± 3.20	24.40 ± 3.12	24.71 ± 2.93	25.04 ± 3.28	25.15 ± 2.78
Water consumption (mL)	20.32 ± 1.14	33.45 ± 1.35*	27.05 ± 2.11*	22.58 ± 1.63^#^	18.04 ± 1.76^#^
Feces number (ea)	41.03 ± 4.52	26.14 ± 3.67*	28.95 ± 2.23*	33.41 ± 3.28^#^	40.01 ± 3.86^#^
Feces weight (g)	4.70 ± 0.41	2.44 ± 0.35*	2.95 ± 0.32*	3.87 ± 0.51^#^	4.98 ± 0.57^#^
Feces water content (%)	58.94 ± 3.28	35.12 ± 1.07*	38.05 ± 1.34*	50.29 ± 2.17* ^,^ ^#^	69.94 ± 2.06^#^

* *p* < .05 compared to the Ctrl group.

^#^ *p* < .05 compared to the LOP group.

### 3.2 Effect of QCT on gastrointestinal transit ratio

**FIGURE 2:**
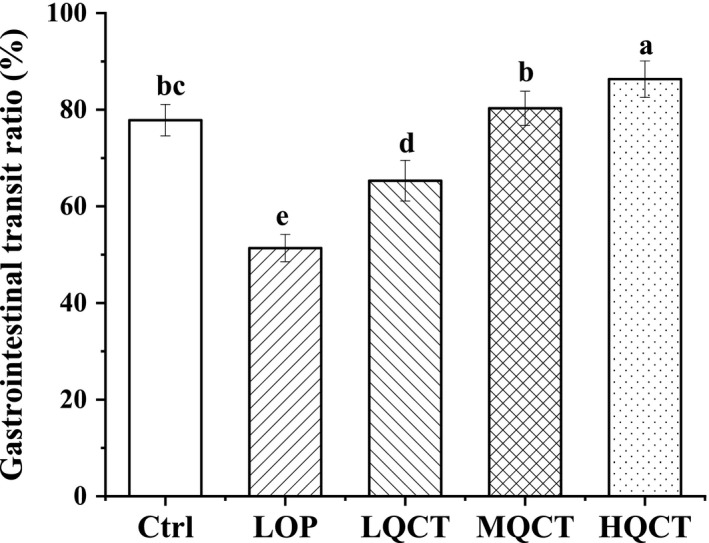
Effect of QCT on gastrointestinal transit of rats. Values are expressed as means ± *SD* of three independent determinations. Means with different letters differ significantly (*p* < .05)

To assess intestinal peristalsis, the gastrointestinal transit ratio was determined. As shown in Figure 2, Lop visibly induced constipation symptoms. However, the transit distance of the charcoal meal was significantly higher in the Ctrl and three QCT groups than that in the LOP group (*p* < .05). The results demonstrated that QCT effectively alleviated the constipation symptoms in rats.

### 3.3 Concentration of short‐chain fatty acids (SCFAs)

**FIGURE 3:**
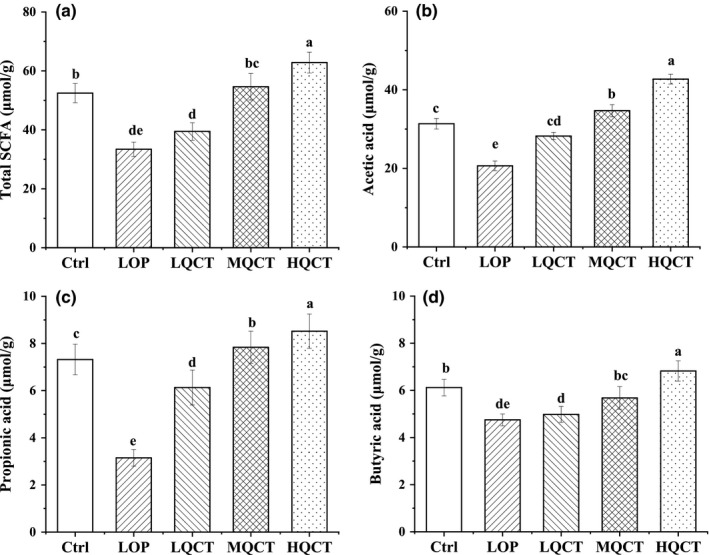
Effects of QCT on the short‐chain fatty levels in the feces of constipated rats. Values are expressed as means ± *SD* of three independent determinations. Means with different letters differ significantly (*p* < .05)

As shown in Figure 3, the concentrations of acetic, propionic and butyric acids, and total SCFA in fecal sample significantly decreased after constipation (*p* < .05). In the LQCT group, the concentrations of butyric acids were no significantly higher when compared to those in the LOP group (*p* < .05). Conversely, the contents of the SCFA markedly increased in rats supplied with the middle and the high dose of QCT when compared with those in the LOP group (*p* < .05).

### 3.4 Effect of QCT on gastrointestinal hormones in blood of rats

**FIGURE 4:**
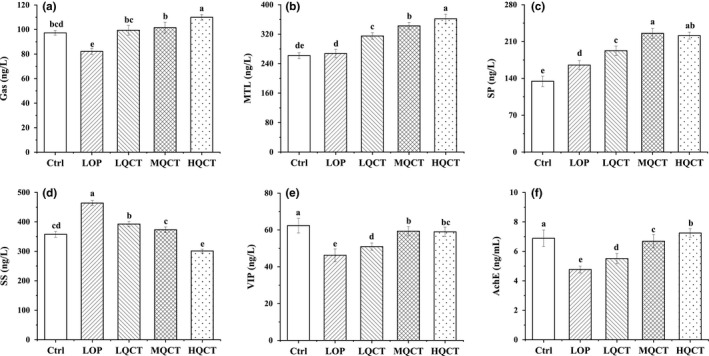
Effect of QCT on serum MTL, Gas, SS, AChE, SP, and VIP levels in rats. Values are expressed as means ± *SD* of three independent determinations. Means with different letters differ significantly (*p* < .05)

The effects of QCT on constipation were further evaluated by measurement of serum parameters in the experimental rats, including MTL, Gas, SP, AchE, SS, and VIP. As shown in Figure 4, in the MQCT and HQCT groups, the levels of MTL, Gas, AchE, VIP, and SP were significantly increased, whereas the levels of SS were significantly decreased (*p* < .05).

### 3.5 Effects of QCT on intestinal flora of constipation rats

**FIGURE 5:**
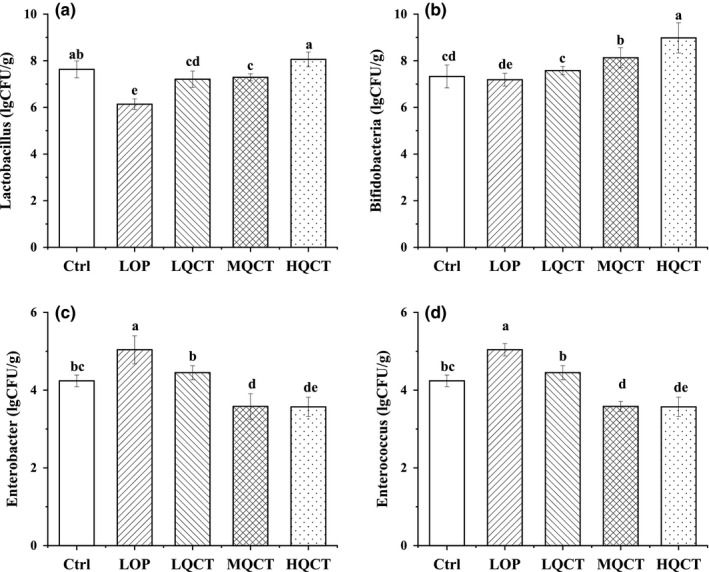
Effect of QCT on intestinal flora of constipated rats. Values are expressed as means ± *SD* of three independent determinations. Means with different letters differ significantly (*p* < .05)

As beneficial intestinal microorganisms, *Lactobacillus* and *Bifidobacteria* can alleviate constipation by breaking down sugar and lowering intestinal pH. The total number of intestinal microorganisms (*Lactobacillus*, *Bifidobacteria*, *Enterobacter,* and *Enterococcus*) in the feces were analyzed (Figure 5). The LOP group given loperamide alone showed the lowest levels of *Lactobacillus* and *Bifidobacteria*. Compared with the LOP group, the number of *Enterobacter* and *Enterococcus* in the feces of rats in the MQCT and HQCT groups was significantly reduced, while the number of *Lactobacillus* and *Bifidobacteria* in the feces of rats was significantly increased. It shows that QCT can inhibit the growth and reproduction of *Enterococcus* and *Enterobacter* in the intestine of constipated rats.

### 3.6 c‐Kit and SCF mRNA expression in small intestine tissue

**FIGURE 6:**
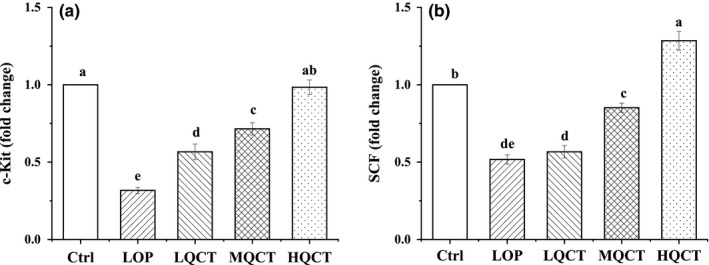
The small intestine tissue levels of mRNA expression of c‐Kit and SCF in QCT treated constipation rats. Values are expressed as means ± *SD* of three independent determinations. Means with different letters differ significantly (*p* < .05)

The small intestine tissue of rats in Ctrl group had the strongest c‐Kit and SCF mRNA (Figure 6), but the rats in LOP group had the weakest expressions. Different doses of QCT could increase these expressions as compared to the LOP group, but the c‐Kit and SCF expressions of rats in HQCT group were both higher than those in MQCT and LQCT groups.

### 3.7 TRPV1, GDNF, AQP3 and iNOS mRNA expression in small intestine tissue

**FIGURE 7:**
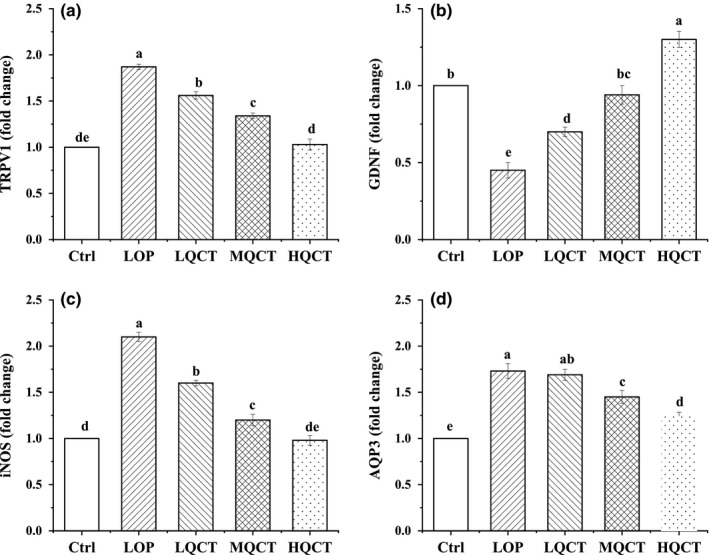
The small intestine tissue levels of mRNA expression of TRPV1, GDNF, AQP3, and iNOS in QCT treated constipation rats. Values are expressed as means ± *SD* of three independent determinations. Means with different letters differ significantly (*p* < .05)

The TRPV1, AQP3, and iNOS mRNA expressions of rats in LOP group were highest, but GDNF expression was lowest (Figure 7). QCT could reduce the TRPV1, AQP3, and iNOS expressions and raise GDNF expression as compared to the LOP group and make these expressions closer to the Ctrl group.

### 3.8 c‐Kit and AQP3 protein expression in small intestine tissue

**FIGURE 8:**
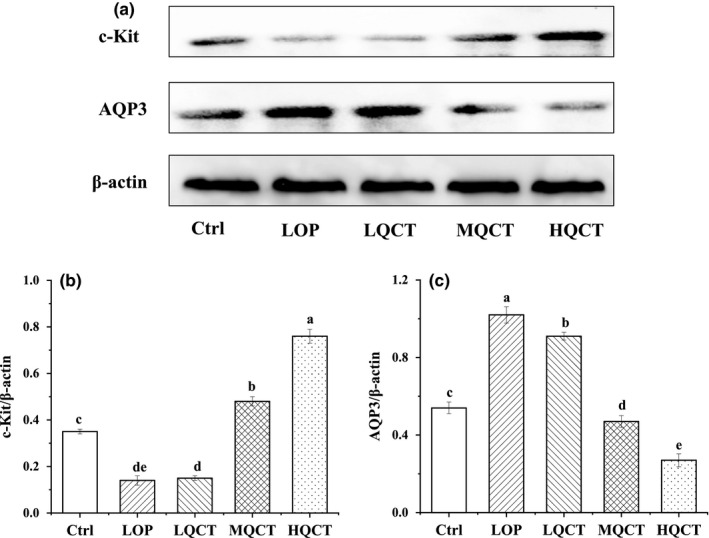
The small intestine tissue levels of protein expression of c‐Kit and AQP3 in QCT treated constipation rats. Values are expressed as means ± *SD* of three independent determinations. Means with different letters differ significantly (*p* < .05)

Since AQPs expression and c‐Kit/SCF signaling pathway play an important role in treating constipation, thus, protein expressions of AQP3 and c‐Kit were detected and analyzed (Figure 8). The results showed that the AQP3 expressions were significantly reduced in QCT groups, while the c‐Kit protein levels were significantly increased. The changes in AQP3 and c‐kit protein expression were similar to those in mRNA expressions.

## 4 DISCUSSION

Constipation affects normal life (such as bloating and loss of appetite) and even causes other intestinal diseases (Zhang et al., 2018). Previous studies have reported that QCT improves constipation, but the underlying mechanisms remain unclear. Loperamide‐induced delay in colonic transit occurs due to inhibition of stool frequency and increased colonic contractions in humans. This drug inhibits intestinal water secretion and colonic peristalsis, which extends the fecal evacuation time and delays intestinal luminal transit (Wintola et al., 2010). The loperamide‐induced rat model of constipation was characterized by decreased fecal pellets, fecal water content, gastrointestinal transit ratio, and fecal SCFA levels, accompanied by an imbalance in intestinal microflora. Therefore, the present study applied loperamide‐induced rat model to explore the possible mechanisms of QCT in improving the secretion of intestinal fluid and gastrointestinal motility. Our data showed that QCT increased the number, weight, and water content of fecal pellets in rats with constipation induced by loperamide, and significantly alleviated constipation. In addition, the laxative effects of QCT are tightly correlated with the interaction between QCT and c‐kit/SCF signaling pathway.

QCT can enhance the number of *Bifidobacteria* and *Lactobacillus*, which are used for production of SCFAs or synthesis of butyric acid from acetic acid. Increased *Bifidobacteria* and *Lactobacillus* counts in feces improve the ratio of beneficial to harmful bacteria in the gut and are involved in increasing the proliferation of beneficial bacteria in the body, thereby improving gut flora. QCT not only affects the composition and quantity of various microbes, but also affects their fermentation products (such as SCFA). SCFAs are the final product of microbial fermentation in the mammalian colon, in which they represent the major organicanions. The ability of the bacteria to produce SCFAs is influenced by the number of bacteria, the pH, and the substrate. The total amount and proportion of SCFAs produced by different substrates differ. SCFAs are involved in important physiological metabolic processes in vivo. Acetic acid could upregulate the barrier function of host intestinal epithelial cells (Fukuda et al., 2011; Jiang et al., 2020). Propionate was thought to reduce fat production, serum cholesterol levels, and carcinogenic effects in other tissues (Hosseini et al., 2011; Jiang et al., 2020). Butyrate had been shown to be a major source of metabolic energy in the large intestine, helping to maintain the integrity of the large intestine, control intestinal inflammation, and support genomic stability (Feng et al., 2005; Jiang et al., 2020). Our study showed that QCT promoted the secretion of SCFAs in constipation rats and increased the contents of acetic acid, propionic acid, and butyric acid to relieve constipation. SCFAs stimulate ileal propulsive contractions by evoking prolonged propagated contractions and discrete clustered contractions (Lan et al., 2020; Wang, Li, et al., 2017; Wang, Hu, et al., 2017). The possible mechanisms by which SCFAs mediate gut motility may involve the intestinal release of 5‐HT (Lan et al., 2020; Robin, 2018). Furthermore, SCFAs could stimulate water and electrolyte absorption, potentiate the proliferation of epithelial cells, influence GI motility, increase mesenteric blood flow, and exert other physiological effects.

QCT not only improved the symptoms of constipation and changed the rat's intestinal flora and SCFAs, but also affected the GI neurotransmitters related to constipation. GI hormones such as MTL, Gas, SP, SS, VIP, and AChE are important indicators of gastrointestinal peristalsis and have been implicated to different extents in normal and pathophysiological situations. MTL, Gas, and SP are excitatory peptide neurotransmitters, whereas SS and VIP are inhibitory peptide neurotransmitters. In this study, the LOP group showed evident constipation symptoms in relation to neurotransmitters, including significantly decreased levels of MTL, Gas, and SP and increased levels of SS and VIP in comparison with the Ctrl group and the three QCT groups. MTL affects the transport of water and electrolytes, stimulate the secretion of pepsin, promotes gastric contractions, and small intestine segmental movement, accelerate intestinal transfer time and improve colon movement (Feighner et al., 1999). Gas stimulates the secretion of gastric acid and pepsinogen and promotes the growth of digestive tract mucosa, the contraction of the GI smooth muscle, and the relaxation of the pyloric sphincter, thus relieving constipation (Suo et al., 2014). SS has been used to stimulate bowel movements and help alleviate constipation (Qian et al., 2018; Soudah et al., 1991). SP adjusts the contraction of the GI tract, intestinal motility, and gastric acid secretion. Therefore, the promotion of the serum levels of MTL, Gas, and SP accelerates intestinal peristalsis and the transport of contents. In our study, the levels of MTL, AChE, Gas, and SP in the QCT group were significantly increased compared with LOP group. These results are consistent with those of several other reports. SS inhibits the release of GI hormones, such as MTL and Gas, and the secretion of gastric acid, trypsin, and amylase. SS could slow the small intestinal transit time significantly, whether during eating or fasting. AChE can regulate muscle contraction and mucus secretion, which can relax the muscles to push the stool out (Furchgott & Zawadzki, 1980; Qian et al., 2018). VIP is a polypeptide composed of 28 amino acids whose function is to relax the GI tract and GI sphincter. VIP is an important factor in the production of descending inhibition, resulting in slow transmission. Studies have found that the levels of SS and VIP in QCT groups were higher than those in LOP group, which means that QCT influenced the level of GI hormones and improved the status of constipation.

Interstitial cells of Cajal (ICC) also affect the gastrointestinal function and acts a pivotal part in the transmission of intestinal nerve signals (Yin et al., 2018). The underlying mechanism is that Low density ICC diminishes the postsynaptic response between ICC and neurotransmitter, leading to the loss of the action of spontaneous rhythmic slowed wave in ICC, irregular colon movement, and influence of the intestinal function (Farrugia, 2008; Liu et al., 2019; Xu et al., 2013; Yu et al., 2016). c‐Kit is the specific marker of ICC and vital to the proliferation of ICC (Brading & Mccloskey, 2005; Liu et al., 2019). SCF regulates the growth and reproduction of ICC. In this study, constipation can reduce the mRNA levels of c‐Kit and SCF in rat small intestine and QCT could effectively upregulate the mRNA levels of c‐Kit and SCF (*p* < .05), which rises the content of ICC in intestine of constipated rat, thus improving constipation. Previous evidence showed that the constipation is tightly correlated with the dysregulation of gastrointestinal motility‐mediating enteric neurons, ICC, and smooth muscle cells (Du et al., 2011; Xin et al., 2018). The neurotransmitters and peptides (such as MTL) released by the enteric nervous system can bind to the receptors on the ICC and promote slow waves generated in ICC to depolarize smooth muscle cells, generating GI contractions, and accelerating intestinal transit. Therefore, we guess that high levels of c‐Kit and SCF promote gastrointestinal hormones (such as MTL, etc.) to improve constipation through the enteric neurons system‐ICC‐smooth muscle signaling pathway. The concrete relationship and mechanism between them still need further research.

TRPV1, a member of the TRPV group of transient receptor potential family of ion channels, is associated with the release of SP. Previous study demonstrated that colonic inflammation triggers the release of pro‐inflammatory neuropeptides SP and CGRP in the urinary bladder via activation of TRPV1 signaling mechanisms enunciating the neurogenic nature of pelvic organ cross‐sensitization. TRPV1 also has been proved to be closely related to defecation. Activation of TRPV1 can trigger neurotransmitter (such as NO) release, resulting in intestinal motility disorder (Qian et al., 2018). Many studies found that reduction expression levels of TRPV1 and NOS in gastrointestinal track could help repair the damaged intestine and prevent constipation (Gan, Ai, et al., 2020; Gan, Liang, et al., 2020; Yin et al., 2018). These research results are consistent with ours. GDNF can regulate the function of ganglion cells, helping repair damaged intestine and prevent constipation (Liu et al., 2019). Constipation is associated with enteric nervous system. NO can relax smooth muscles and slow down bowel movements via inhibiting neurotransmitter of enteric nervous system. Reducing NO content by controlling iNOS is a feasible method to control constipation (Boots et al., 2008; Liu et al., 2019). Regulating the expression levels of TRPV1, GDNF, and iNOS to relieve constipation is one of the mechanisms underlying the inhibitory role of QCT in constipation.

AQPs can affect the changes in colonic water metabolism and intestinal permeability (Deng et al., 2018; Zhan et al., 2020). Water is transported from the luminal side to the vascular side through AQP3 because the osmotic pressure is lower in the colonic lumen than in the blood vessel, and the feces are concentrated. Too much water is absorbed from the intestinal contents when the AQP3 expression level increases. Consequently, feces within the colon harden and aggregate, which leads to difficult defecation and constipation (Risako et al., 2015). Gastrointestinal hormone VIP plays an important role in water transport in the intestine (Yi et al., 2019). VIP, one of the most abundant neuropeptides in the intestine, has been shown to play an important role in water transport by regulating the cAMP‐PKA‐AQP signal transduction pathway (Gan, Ai, et al., 2020; Gan, Liang, et al., 2020; Zhan et al., 2020). In this study, we found that QCT can downregulate the expressions of AQP3 mRNA and protein via VIP‐cAMP‐PKA‐AQP3 signaling pathway, which prevent the reabsorption of water in the lumen by blood vessels, thus improving constipation.

In conclusion, by measuring related constipation indicators (stool status, water content of defecation, GI transit rate, and so on), it was found that QCT could alleviate constipation. The serum levels results showed that QCT could improve the content of MTL, GAS, AChE, SP, and VIP and lower the level of SS in the constipated rats. RT‐qPCR and/or Western blot experiments further showed that QCT could upregulate the mRNA and/or protein expressions of c‐Kit, SCF, and GDNF, and downregulate the expression of TRPV1 and NOS in constipated rats. These findings further indicate that QCT could be considered a potential therapeutic candidate for the treatment of constipation, although many additional studies are required to confirm the above.

## CONFLICT OF INTEREST

The authors declare that they have no competing interests.

## ETHICAL APPROVAL

This article does not cover any human studies conducted by any of the authors. All animal experimental procedures were approved by the Institutional Animal Care and Use Committee at Jiangnan University (No. No20180115b1920520).
